# Perceived Economic Uncertainty and Fertility Intentions in Couples: A Dyadic Extension of the Theory of Planned Behaviour

**DOI:** 10.1007/s10834-022-09872-x

**Published:** 2022-11-01

**Authors:** Camilla Matera, Lars Dommermuth, Silvia Bacci, Bruno Bertaccini, Alessandra Minello, Daniele Vignoli

**Affiliations:** 1 Department of Education, Languages, Intercultures, Literatures and Psychology (FORLILPSI), University of Florence, Via Di San Salvi, 12–Pad. 26, 50135 Florence, Italy; 2 Research Department, Statistics Norway, Pb 2633 St. Hanshaugen, 0121 Oslo, Norway; 3 Department of Statistics, Computer Science, Applications “G. Parenti” (DISIA), University of Florence, Viale Morgagni 39, 50134 Florence, Italy

**Keywords:** Fertility, Theory of planned behavior, Attitudes, Intentions, Dyadic perspective, APIM

## Abstract

By adopting a dyadic extension of the Theory of Planned Behavior (Ajzen, 1991), this study examined whether perceived economic uncertainty affects fertility intentions. Three-hundred thirty one heterosexual couples living in Italy participated in a randomized between-group experimental study, in which we manipulated perceived economic uncertainty (low vs. high vs. control). The participants subsequently completed a questionnaire measuring their attitudes, subjective norms, perceived behavioral control, and fertility intentions. We employed Structural Equation Modelling in estimating the Actor–Partner Interdependence Model. The model showed a good fit to the data. Women’s attitudes, subjective norms, and perceived behavioral control were influenced by the high economic uncertain scenario, whereas among men these variables were affected only by the positive economic scenario. Attitudes and perceived behavioral control were significant predictors of fertility intentions for both sexes. Significant partner effects were observed as well. These findings suggest that fertility plans should be examined by adopting a dyadic perspective, as individuals’ intentions are affected not only by their own beliefs, but also by those of their partners.

## Introduction

Over the past decades, most European countries have experienced a decline in fertility rates, which accelerated in the aftermath of the Great Recession in 2009. Italy, for example, has experienced a constant fertility decline since 2010, with an average number of children per women (total fertility rate) of 1.29 in 2019 (Istat, 2020). This trend was highly unexpected among social observers (Goldstein et al., 2009), and led to a renewed interest in understanding childbearing decisions in Europe.

In most modern societies, reproduction is often the result of a reasoned decision-making process. With easy access to contraceptives and legally-guaranteed abortion rights, the birth of a child tends to be a voluntary and reasoned decision based on a cost–benefit analysis (Ajzen & Klobas, 2013; Thomson & Brandreth, 1995). In Italy, much research has confirmed that reproduction is the consequence of a reasoned process. First, rates of unintended pregnancies—including among adolescents—are extremely low (Castiglioni et al., 2001; Sedgh et al., 2011). Second, fertility outcomes are highly consistent with previously stated intentions; in particular, negative fertility intentions have been shown to almost perfectly predict subsequent realizations (Régnier-Loilier & Vignoli, 2011).

Fertility decisions in many European countries are often made under conditions of rising economic uncertainty, in which the future becomes less predictable. To date, much demographic research has examined economic factors affecting fertility intentions (e.g., Busetta et al., 2019; Hanappi et al., 2017; Modena et al., 2013; Sinyavskaya & Billingsley, 2015; Vignoli et al., 2013, 2020a, 2020b). However, deeper analyses into the socio-psychological processes involved in forming childbearing intentions remain scarce, especially in Italy. Moreover, evidence is exiguous on the potential mutual influence between partners regarding the direct antecedents of fertility intentions, and it is still unclear whether interpersonal factors, together with intrapersonal ones, can be responsible for the formation of individuals’ fertility intentions.

Through the present study, we aimed to address these oversights in previous research by examining how perceived economic uncertainty affects the fertility intentions of both members of a couple, and by proposing a dyadic extension of one of the most used psychosocial frameworks for understanding fertility choices: The Theory of Planned Behavior (TPB; Ajzen, 1991).

## The Theory of Planned Behavior

The TPB is one of the most frequently cited and influential models for the prediction of human social behavior (Ajzen, 2011). The TPB has also emerged as one of the dominant models in fertility research in recent years (Brehm & Schneider, 2019). Indeed, demographers and social scientists have increasingly applied it to the investigation of childbearing decision-making processes (e.g., Ajzen & Klobas, 2013). In this model, behavioral intentions are defined as a plan or a likelihood that the individual will act in a particular way in a specific situation, and a given context and timeframe, meaning that intentions are the best predictor of effective behavior (Ajzen, 1991). In line with the model, different studies have found that fertility intentions are effective predictors of subsequent fertility behavior (see, for example, Dommermuth et al., 2015; Mencarini et al., 2015; Testa & Toulemon, 2006).

Intention formation is based on a set of beliefs that form three determinants: attitudes, subjective norms, and perceived behavioral control (Ajzen, 1991). Attitudes derive from behavioral beliefs, which are the perceived positive or negative consequences of the behavior (Fishbein & Ajzen, 1975). In the current study, this includes beliefs concerning the consequences of having, or not having, a child. Subjective norms derive from perceived expectations of important referents concerning said behavior, combined with the motivation to comply with them (Ajzen & Fishbein, 1980). This includes, for example, individuals’ perceptions that their parents or friends would (dis)agree with their decision to have a child. Indeed, several studies have shown that family events are facilitated by societal, familial, and individual approval in one’s social surroundings (Kalmijn, 2004). In a country characterized by strong family ties (Reher, 1998), such as Italy, individuals are especially likely to feel parental pressure on family decisions (Dalla Zuanna & Micheli, 2004)—such as having a child (Dalla Zuanna & Micheli, 2004; Livi Bacci, 2001)—and to weigh the decision to adopt a new living arrangement with their family of origin’s level of acceptance (Rosina & Fraboni, 2004). Not only parents, but also the voices of friends and peers have been suggested as increasingly important in qualitative studies on family life in Italy (Vignoli & Salvini, 2014). Consistently, in a quantitative study conducted among Italian men and women (Baroni et al., 2019), one’s parents, friends and partners emerged as important with respect to the fertility intentions of both sexes—although friends were considered as less important than parents, and parents less important than partners.

Finally, perceived behavioral control refers to the perception of being able to perform the behavior in question, based on the consideration of both internal and external constraints and/or resources (Ajzen, 1991; Ajzen & Madden, 1986). This may include the degree to which individuals perceive they are able to care for a child, especially when considering their general life or employment situation.

Ajzen and Klobas (2013) investigated the role of attitudes, subjective norms, and perceived behavioral control in predicting fertility intentions in a comparative perspective including five European countries. Their results showed that all three factors significantly impacted intentions (except for subjective norms in France, and perceived behavioral control in Italy and Germany). Furthermore, childbearing attitudes were the factor with the strongest impact on intentions in Russia, Italy, and Hungary, while in France attitudes and perceived behavioral control were equally strong, and subjective norms had the strongest effect in Germany (Ajzen & Klobas, 2013). Other studies focusing on single countries confirmed that all three factors, or specific combinations thereof, had a significant impact on fertility intentions (see, for example, Billari et al., 2009 for Hungary; Dommermuth et al., 2011 for Norway; or Mencarini et al., 2015 for Italy).

## Background Factors: The Role of Economic Uncertainty

What contributes to shaping an individual’s attitudes, subjective norms and perceived control over one’s behavior? Background factors could be especially relevant in influencing these psychological processes. According to the TPB, these background factors can directly influence intentions by affecting the beliefs that shape attitudes, subjective norms, and perceived behavioral control (Ajzen, 2011). Most of the existing research on fertility intentions has highlighted the importance of economic factors associated with the formation of fertility intentions and decisions, although often excluding the TPB-related psychological antecedents of intentions from their analyses (e.g., Busetta et al., 2019; Hanappi et al., 2017; Modena et al., 2013; Vignoli et al., 2020a, 2020b). Previous research has operationalized economic uncertainty, using objective indicators of individuals’ labor-market situation, such as temporary contracting or unemployment (Kreyenfeld, 2010, 2015; Kreyenfeld et al., 2012; Mills & Blossfeld, 2013; Vignoli et al., 2012), or such macro-level indicators as unemployment rates or gross-domestic product (Adsera, 2005; Comolli et al., 2020; Hoem, 2000; Kravdal, 2002; Sobotka et al., 2011). However, the impacts of such indicators may differ by setting; for example, in some countries, having a job has a positive impact on fertility intentions, while in other countries women out of the labor market are more likely to intend to have a child (Fahlén, 2013). This may be due to the same background factor being differently perceived by individuals based on their cultural background. Indeed, some previous studies have suggested that individuals’ perceptions of economic uncertainty may differ from objective uncertainty. For instance, Hofmann and Hohmeyer (2013) found that strong economic concerns were associated with lower fertility among German women who were cohabiting with a male partner. Sobotka et al. (2011) pointed out that apprehension regarding future negative economic events might shape fertility; the way in which individuals perceive their broader economic climate may increase uncertainty, and in turn influence fertility.

## Perceived Economic Uncertainty and Fertility Intentions

In conditions of mounting uncertainty, as in Italy, men and women appear to be more careful in their childbearing planning (Novelli et al., 2021). According to Vignoli et al., (2020a, 2020b), individuals’ childbearing decision-making could be largely affected by the way they envisage their future, especially under conditions of uncertainty; in this case, individuals are more likely to make decisions that may be independent from their actual economic situation and constraints. Thus, fertility intentions seem to be influenced not only by actual structural limitations, but also by how individuals subjectively perceive their own situation and the broader economic climate. Despite the importance of considering such a subjective perspective, the link between perceived economic uncertainty and fertility intentions has been somewhat neglected. An exception to this is an experimental study conducted in Italy and Norway by Vignoli et al. (2022), in which participants were presented with mock newspaper stories describing either a positive or negative hypothetical future economic scenarios. This experimental evidence confirmed that fertility intentions were affected by shared narratives of the future in both countries (Vignoli et al., 2022). However, Vignoli et al. (2022) adopted an individual level of analysis, meaning that they did not clarify whether perceived economic uncertainty might impact both partners’ fertility intentions in the same way. Moreover, they did not apply the TPB to examine fertility intentions, so that the role of psychological factors in response to hypothetical future economic scenarios has yet to be made clear. We address this research gap by conducting an experimental study in which perceived economic uncertainty was manipulated (Vignoli at el., 2022), but a dyadic approach and a psychosocial perspective were adopted, thus allowing us to assess the fertility intentions of the two partners of a couple, together with their most relevant psychological antecedents.

## A Couple Perspective: A Dyadic Extension of the TPB

The TPB has been supported by research in many domains (Armitage & Conner, 2001). Intentions represent the motivation to act and, if appropriately measured, can account for an appreciable proportion of variance in actual behavior (Ajzen, 1991). Nevertheless, there are circumstances in which intentions are insufficient for prompting action. This happens when a behavior cannot be completely deliberative, as it does not exclusively depend on an individual’s intention to act. There are many reasons why this might be the case. For instance, an intention may not materialize when the behavior depends not only on one individual’s willingness to engage in it, but also on the willingness of another person (Ajzen, 1991).

It is undeniable that reproductive behavior is not exclusively under an individual’s volitional control. Although fertility intentions have been found to be effective predictors of individual childbearing behavior (Schoen et al., 1999; Testa & Toulemon, 2006), the choice to have a child is most often the result of a dyadic process in which both partners exert influence (Stein et al., 2014). In other words, in advanced societies, childbearing is generally the outcome of a joint couple decision-making process (Duvander et al., 2020). The partner’s fertility intentions play an important role in the realization of an individual’s intention (Thomson et al., 1990). Both partners have a central role in affecting the outcome of such decision-making (Becker, 1996). Although it could seem obvious that having a child depends on the desires and plans of two individuals, many studies in this field have typically examined fertility intentions using an individual level of analysis. Even when recognizing the importance of using couple data, many studies have typically relied on women’s reports, without examining their male partners’ intentions directly (Stykes, 2018; Waller & Bitler, 2008). Mainly due to the difficulties of collecting data from both members of a couple, comparatively few studies have examined both parents’ reported fertility intentions. By including both partners into their analyses, Li et al. (2019) identified different constellations of paternal and maternal fertility intentions, showing that they are often configured in heterogeneous ways. Among stable couples in Sweden, the intentions of both partners was important for the realization of positive fertility intentions, and especially for the intention to become a parent for the first time (Duvander et al., 2020), thus supporting previous results from the same country (Thomson & Hoem, 1998). A German-based study showed symmetrical effects of both partners’ desires for children (Bauer & Kneip, 2013). In contrast, a study employing couple data from the US reported that women’s desires were associated both with first and subsequent births, while men’s desires were only indirectly associated with the latter (Ray et al., 2020). In a study about couples’ childbearing behavior in Italy (Testa et al., 2011), women were found to have a stronger effect on fertility decisions than their male partners. Although these studies used dyadic data to analyze fertility intentions, mutual influences between partners within the dyad were not examined. Matias and Fontaine (2017) analyzed dyadic associations between motivations toward parenthood (e.g., social recognition, emotional enrichment) and fertility intentions in dual-earner couples. Their study highlighted the importance of adopting a dyadic perspective that considers mutual influences between partners. Apart from this single study, there is scant evidence on whether there is a mutual influence between partners regarding the direct antecedents of their individual fertility intentions. Based on the above, we propose a dyadic extension of the TPB, which considers the attitudes, subjective norms, perceived behavioral control, and intentions of both relationship partners to determine whether these theoretical constructs operate through interpersonal as well as intrapersonal channels. Such a dyadic model was proposed and tested in one previous study concerning the health behaviors of parents and their adolescent children (Lenne et al., 2019), which enabled the researchers to disentangle effects that were uniquely interpersonal from those that were intrapersonal. The application of this model to the fertility domain might be especially important if we consider that planning the birth of a child is something intrinsically linked to the couple, more so than it might be for behaviors that, although potentially influenced by interpersonal channels, are not fundamentally a couple matter, such as health habits.

A couple perspective is absent in *TPB-based* fertility research, which could well be related to the model itself, as the TPB primarily aims to understand *individual* decision making (Ajzen, 1991). Discussing the TPB’s application in fertility research, Ajzen and Klobas (2013) claimed that a couple perspective, or “the partner,” is already embedded in the existing model either as an important normative referent (subjective norms) or through indicators for perceived behavioral control (e.g., by questions asking whether a partner’s work may interfere with one’s fertility intentions). However, they recognized in a footnote that fertility decisions typically involve two people, as a partner who refuses to have a child usually constitutes a significant barrier to childbearing (Ajzen & Klobas, 2013). Taking full advantage of couple data, we seek to investigate the possible reciprocal influences between partners when they form their own fertility intentions in an Actor–Partner Interdependence Model (APIM; Kenny, 1996).

## Hypotheses

To the best of our knowledge, no previous studies have examined both partners’ fertility intentions, nor their predictors, in the framework of the TPB. As such, we aimed to bridge this gap by examining how perceived economic uncertainty—which could be perceived differently by each member of a couple—can affect individuals’ and their partners’ fertility intentions. Notably, we adopted a dyadic perspective, through which we considered not only the fertility intentions, and their direct predictors, of both partners (i.e., their attitudes, subjective norms, and perceived behavioral control), but also the reciprocal influences within the dyad. Through an experimental study design, in which perceived economic uncertainty was manipulated (Vignoli et al., 2022), we tested the following hypotheses.

### Hypothesis 1

(H1) Perceived economic uncertainty will affect the attitudes, subjective norms, and perceived behavioral control of both partners. Low perceived economic uncertainty will likely produce more positive attitudes, more favorable subjective norms, and higher perceived behavioral control, while high perceived economic uncertainty will generate more negative attitudes, less favorable subjective norms, and lower perceived behavioral control.

### Hypothesis 2

(H2) An individual’s attitude, subjective norms, and perceived behavioral control will predict not only their intention to have a child, but also that of their partner.

### Hypothesis 3

(H3) The relationship between economic uncertainty and fertility intentions of both couple members will be mediated by the two partners’ attitudes, subjective norms and perceived behavioral control.

## Methods

### Design and Procedure

We conducted a laboratory experiment at the university with which some of the authors are affiliated. The experiment took place across different sessions between June 2019 and early February 2020, and was implemented using the O-TREE open-source platform (Chen et al., 2016). We used a randomized between-groups experimental design. The independent variable was perceived economic uncertainty, which was manipulated on three levels: we had two experimental conditions (low vs. high economic uncertainty) and one control group. The participants were randomly assigned to one of these three conditions. We manipulated perceived economic uncertainty by presenting participants with mock newspaper stories describing either a positive or negative hypothetical future economic scenario (see Appendix). We chose to present the scenario as a newspaper article, as the press (including online newspapers) is considered very or fairly reliable by a significant proportion of Italians (64,3%; Censis, 2018). In a pilot study conducted prior to the experiment, similar scenarios to the ones used in the present study were evaluated as realistic by a sample of 200 participants (M = 5.25, SD = 1.18 on a 7-point Likert scale).

In line with previous experimental studies using scenarios (e.g., Fornaini et al., 2021; Matera, 2014; Matera et al., 2018), we opted for a between-subjects design, presenting only one vignette to each respondent, thus making it impossible for them to make comparisons between the different scenarios and answer accordingly, which could introduce some forms of bias. Moreover, such a design avoided fatigue in the respondents, given the relatively long mock news story used as a treatment. In the positive and negative scenarios, three economic aspects anticipated over the following three years were described: jobs with uncertain conditions (juxtaposition of permanent and temporary jobs); instability of professional careers (whether young people will secure a stable position or not); and joblessness (chances to find or to lose a job). Participants in the control condition were not exposed to any scenario.

Only couples were invited to participate in the study, and both members participated at the same time. Each member of a couple completed the questionnaire individually and at the same time, separated from the other in different rooms. Furthermore, the participants were not informed about the scope of the study beforehand so that partners could not discuss their fertility intentions with each other before arriving at the laboratory.

The sample size was determined before any data analysis. A power analysis using G*Power (Erdfelder et al., 1996) indicated that a minimum sample of 135 would be needed to detect small to medium effects (Pillai V = 0.20), with 95% power using a multivariate analysis of variance (MANOVA) with alpha set at 0.05. Moreover, for testing complex models with internally consistent and highly interrelated indicators, a minimum sample size of 200 is recommended (Weston & Gore, 2006). Our sample of 662 participants thus fulfilled these criteria. Participants were recruited through the services of specialized survey agencies. Regarding the inclusion criteria, we only included couples living in Italy who had been together for over 4 months; moreover, women had to be aged between 20 and 40. We ensured a balanced participation of jobless, permanently employed, and temporarily employed women. Additionally, the respondents were recruited in such a way as to have a balance between those with and without children. The participants were given an economic incentive for their participation, which consisted of 50 euros per couple. The protocol was approved by the Ethics Committee of the University of Florence.

### Participants

The participants were 331 heterosexual dating couples, either living apart together (LAT), cohabiting, or married (662 individuals). The duration of couples’ relationships varied: 6.35% had been in their relationship for 1 year or less, 26.32% for 2–5 years, 32.67% for 6–10 years, and 34.66% for over 10 years. A majority (44.90%) were cohabitants, 32.12% were married, and 22.98% were LAT. Almost half of the couples did not have children (50.91%), while 30.18% had one child, 15.24% had two children, and a small percentage (3.66%) had three or more children. Women ranged in age from 20 to 40 years (M = 32.39, SD = 5.08), while men were aged between 20 and 53 (M = 35.12, SD = 6.74). Most men had a permanent job (53.50%), 29.48% had a temporary job, and 17.02% were jobless. As for the female participants, 36.47% had a permanent job, 30.70% had temporary work, and 32.3% did not work. In terms of educational attainment, 51.06% of men had completed secondary education, 33.43% had a Bachelor’s, Master’s, or Ph.D. degree, while 15.50% had completed primary education. Among women, 57.93% had completed secondary education, 34.76% had a Bachelor’s, Master’s, or Ph.D. degree, while 7.32% had completed primary education.

### Measures

After reading the newspaper story depicting either a positive or negative hypothetical future economic scenario, the participants were asked to imagine themselves in the described scenario and complete a questionnaire containing several measures. Some of these were not relevant to the hypotheses tested in the present study, and will thus not be reported here (all of them were presented after the measures we used in the present study—open questions about uncertainty, lotteries/batteries to measure time preferences and risk aversion, Raven matrices test (selection) and logic test).

As suggested by Ajzen and Klobas (2013), when measuring attitudes, subjective norms, and perceived behavioral control as predictors of fertility intentions, it is important to define the precise behavioral goal of the intention being assessed. We considered “Having a child during the next three years” as our fertility-related goal. Questions regarding intentions “in close temporal proximity to the prospective behavior” (Ajzen & Fishbein, 1973, p. 49) have been documented as fairly suitable predictors of actual behavior by several demographic studies (e.g., Philipov, 2009; Régnier-Loilier & Vignoli, 2011; Speder & Kapitany, 2009). Although most studies in demography used a single-item scale to assess fertility intentions (Lappegård et al., 2021; Neyer et al., 2013), we preferred to use two items to assess this construct, in order to increase the reliability of our index.

All the scales used in the present study had been proven to be valid and reliable in a previous study on fertility intentions among Italian women and men (Baroni et al., 2019). Nevertheless, we again examined their convergent validity and reliability by estimating composite reliability (CR), the average variance extracted (AVE), and Cronbach’s alpha for each scale, respectively, among men and women. Given that fertility intentions were assessed using two items, the Spearman-Brown coefficient was computed as well (Eisinga et al., 2013). The results of these analyses are presented in Table 1.

**Table 1:** Measurement part of the estimated SEM—Factor loadings, average variance extracted (AVE), composite reliability (CR) and Cronbach’s alpha

	Factor loadings (Unstandardized coefficients)	SE	*z-*value	*P(* > *|z|)*	Factor loadings (Standardized coefficients)	Composite reliability	Average variance extracted (AVE)	Cronbach's alpha
Women								
Intention						0.89	0.80	0.90
Item 1	1				0.880			
Item 2	0.947	0.042	22.428	< 0.001	0.913			
Attitude						0.92	0.62	0.92
Item 1	1				0.932			
Item 2	0.743	0.033	22.575	< 0.001	0.830			
Item 3	0.779	0.040	19.54	< 0.001	0.776			
Item 4	0.997	0.036	27.73	< 0.001	0.898			
Item 5	0.661	0.048	13.845	< 0.001	0.635			
Item 6	0.660	0.034	19.215	< 0.001	0.770			
Item 7	0.596	0.045	13.259	< 0.001	0.617			
SN						0.75	0.50	0.78
Item 1	1				0.742			
Item 2	0.772	0.074	1.400	< 0.001	0.673			
Item 3	0.826	0.077	1.666	< 0.001	0.696			
PBC						0.86	0.67	0.86
Item 1	1				0.767			
Item 2	1.357	0.079	17.115	< 0.001	0.908			
Item 3	1.061	0.073	14.559	< 0.001	0.775			
Men								
Intention						0.86	0.75	0.87
Item 1	1				0.832			
Item 2	0.994	0.053	18.663	< 0.001	0.903			
Attitude						0.91	0.60	0.91
Item 1	1				0.916			
Item 2	0.678	0.034	2.186	< 0.001	0.803			
Item 3	0.768	0.038	19.995	< 0.001	0.799			
Item 4	0.913	0.039	23.645	< 0.001	0.864			
Item 5	0.589	0.047	12.442	< 0.001	0.598			
Item 6	0.650	0.037	17.564	< 0.001	0.746			
Item 7	0.617	0.046	13.35	< 0.001	0.628			
SN						0.71	0.44	0.73
Item 1	1				0.720			
Item 2	0.661	0.076	8.758	< 0.001	0.583			
Item 3	0.814	0.083	9.774	< 0.001	0.674			
PBC						0.84	0.63	0.83
Item 1	1				0.698			
Item 2	1.433	0.102	14.062	< 0.001	0.892			
Item 3	1.113	0.086	12.943	< 0.001	0.786			

The Spearman-Brown coefficient for the two items assessing intention was 0.82 for women and 0.76 for men (p < .001)

#### Fertility Intentions

The intention to have a child in the next three years was assessed using two items (e.g., “Do you intend to have a child in the next three years?” and “How happy would having a child in the next three years make you?”). Responses were given on an 11-point Likert scale, from 0 (*definitely not)* to 10 (*definitely yes*). Higher scores indicated higher fertility intentions.

#### Attitudes

The attitudes toward having a child in the next three years were assessed by seven semantic differential items measured on a 5-point scale (i.e., “Having a child in the next three years would be: negative–positive, bad-good, unpleasant-pleasant, undesirable-desirable, foolish-wise, disagreeable-agreeable, dangerous-safe”). High scores indicated more favorable attitudes.

#### Subjective Norms

Subjective norms were assessed by asking the participants to indicate the extent to which some significant others (i.e., parents, friends, partner) would approve if they decided to have a child in the next three years. The responses were provided on a 5-point Likert scale from 1 (*completely disagree*) to 5 (*completely agree*). Higher scores indicated more favorable subjective norms.

#### Perceived Behavioral Control

The perception of the control over one’s behavior was measured using three items (e.g., “It would be easy for me to have a child”) rated on a 5-point Likert scale from 1 (*completely disagree*) to 5 (*completely agree*). Higher scores indicated higher perceived behavioral control.

#### Manipulation Check

As part of our manipulation check, the participants were asked to indicate the valence of the scenario they were presented with on a 5-point scale from 1 (*a definitively negative scenario*) to 5 (*a definitively positive scenario*). Those in the control group did not answer this question, as they were not presented with any story depicting a future scenario.

### Data Analyses

**Fig. 1:**
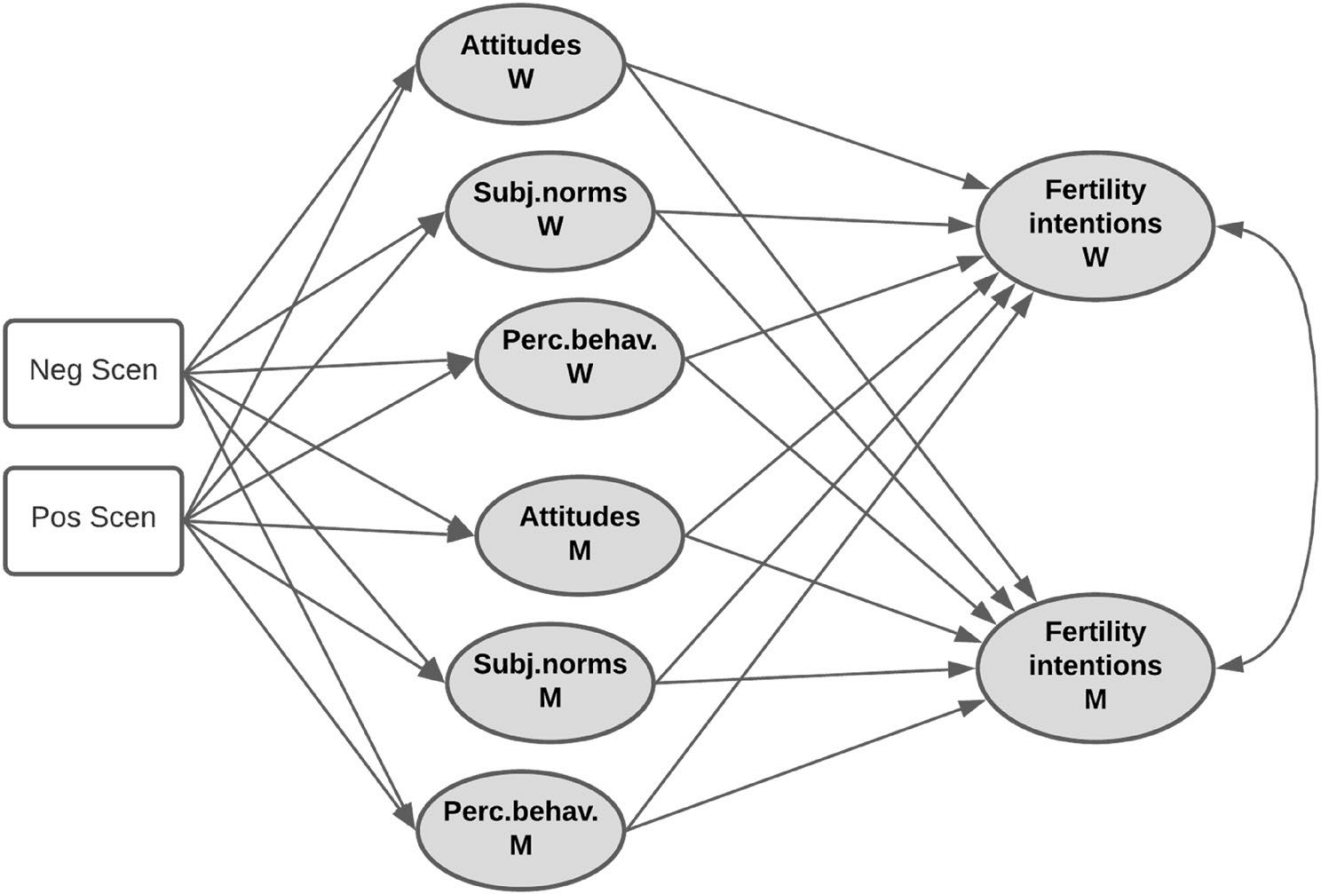
Structural part of the theoretical SEM (variances and covariances omitted for the sake of parsimony)

A MANOVA was performed to test the effect of the independent variable (economic uncertainty) on the manipulation check among women and men separately. Correlations among variables were calculated separately for the two members of the couples. We used the Actor–Partner Interdependence Model (APIM; Kenny, 1996) to test our hypotheses from a couple perspective. The APIM is a model of dyadic relationships that considers the interdependence in two-person relationships. One critical distinction in dyadic research is whether members of dyads are distinguishable; in this case, the variable gender allowed us to distinguish between the female and male members of the dyad. The APIM’s two central components are actor effects and partner effects. In our model, actor effects measure how much an individual’s fertility intentions are predicted by their own attitudes, subjective norms, and perceived behavioral control. Partner effects measure how much an individual’s fertility intentions is influenced by their partner’s attitudes, subjective norms, and perceived behavioral control. Notably, actor effects are estimated controlling for partner effects, and vice versa. We employed Structural Equation Modelling (SEM; Bollen, 1989; Bollen et al., 2008; Duncan, 1975) in estimating the APIM (Kenny & Ledermann, 2010). In the present contribution, all the involved latent variables were endogenous, since they were affected by other latent or observed variables. Indeed, fertility intention was affected by attitudes, subjective norms, and perceived behavioral control, which were in turn affected by the economic scenario. The economic scenario is an exogenous observed variable defined according to the two experimental (negative and positive scenario) groups, and compared against the control group (i.e., reference category). The structural part of the theoretical SEM is displayed in Fig. 1 (the measurement part has been omitted for the sake of clarity).

In SEM approaches, sampled data are usually assumed to follow a multivariate normal distribution, so that the vector of the means and the matrix of covariance contain all the information required for the estimation procedure. The most widely used estimation method is the Maximum Likelihood (ML). When data are non-normal (e.g., ordinal), some alternative estimation procedures can be used based on the weighted least squares fit function (see, for instance, Muthén, 1984; Muthén & Asparouhov, 2003; Muthén, 2004). However, when data are ordinal, the categorical nature of the variables can be ignored, providing that the number of categories is at least five and the data show an approximately normal distribution (Bollen, 1989). We followed this last approach, thus using the ML estimator. Estimates were performed with the R package lavaan, version 0.6–8 (Rosseel, 2012).

Regarding the convergent validity and reliability of our measures, an AVE of 0.50 or above and a CR of greater than 0.70 were considered as compelling demonstrations of convergent validity (Fornell & Larcker, 1981; Hair et al., 2009). We also considered AVE values of above 0.40 acceptable if the CR was higher than 0.70 Lam (2012). For Cronbach’s alpha, values of 0.70 or above were deemed as acceptable (Cortina, 1993).

The evaluation of the model fit was driven by the Tucker and Lewis Index (TLI; Tucker & Lewis, 1973), the comparative fit index (CFI; Bentler, 1990), and the root mean square error of approximation index (RMSEA). These indices integrate the information about the model fit obtained from the chi-square test, the reliability of which is strongly affected by the sample size. Indeed, for small sample sizes, the test can suggest accepting the model even if its fit is poor; conversely, when the sample size is overly large, the test could incorrectly suggest rejecting the model. We should note that all fit indices have limitations, so that using a combination provides a more comprehensive sense of model fit than a single index (Tabachnick & Fidell, 2007). For CFI and TLI, values equal to or greater than 0.90 denote a good fit (Bentler, 1990; Byrne, 1998). A RMSEA lower than 0.05 indicates a good fit, while a value between 0.05 and.08 indicates a reasonable fit (Byrne, 1998). We examined the mediated relationships in the model using indirect effects in the final model.

## Results

### Preliminary Analyses

Descriptive statistics for all variables in the three experimental conditions are reported in Table 2.

**Table 2:** Descriptive statistics: Means (standard deviations in parentheses) for manipulation of perceived economic uncertainty

Experimental condition	Women				Men				N (couples)
	Attitude	SN	PBC	Intention	Attitude	SN	PBC	Intention	
Control	3.42 (1.01)	3.90 (0.96)	3.09 (1.10)	5.35 (3.31)	3.60 (1.06)	3.82 (1.00)	3.30 (1.20)	5.45 (3.50)	109
High economic uncertainty	2.98 (0.89)	3.37 (0.99)	2.52 (1.07)	4.37 (3.08)	3.43 (0.92)	3.63 (0.94)	2.98 (1.17)	5.06 (3.11)	112
Low economic uncertainty	3.98 (0.89)	4.09 (0.90)	3.53 (1.24)	7.33 (3.02)	4.06 (0.77)	4.12 (0.78)	3.63 (1.05)	7.09 (3.11)	110

First, we performed a MANOVA to test the effect of the independent variable (economic uncertainty) on the manipulation check for both women and men. Perceived economic uncertainty was successfully manipulated: we found a main effect of the scenario on our manipulation check for both women (F_(1, 220)_ = 1166.73, p < 0.001, η^2^ = 0.84) and men (F_(1, 220)_ = 442.43, p < 0.001, η^2^ = 0.67); (Wilks λ = 0.13, F_(2, 219)_ = 739.41, p < 0.001, η^2^ = 0.87). In both cases, the scenario was evaluated as more positive (M_W_ = 4.20; M_M_ = 4.30) in the low, rather than high (M_W_ = 1.41; M_M_ = 1.82), economic uncertainty condition. We then calculated correlations among the TPB variables (see Table 3).

**Table 3:** Correlations between variables (W = women, M = Men)

	1	2	3	I4	5	6	7
1. Attitude—W	–						
2. SN—W	0.581***	–					
3. PBC—W	0.746***	0.550***	–				
4. Intention—W	0.787***	0.475***	0.691***	–			
5. Attitude—M	0.469***	0.352***	0.427***	0.505***	–		
6. SN—M	0.421***	0.288***	0.363***	0.408***	0.707***	–	
7. PBC—M	0.501***	0.386***	0.379***	0.478***	0.571***	0.498***	–
8. Intention—M	0.488***	0.380***	0.463***	0.550***	0.750***	0.605***	0.528***

### SEM Estimation and Fitting

**Fig. 2:**
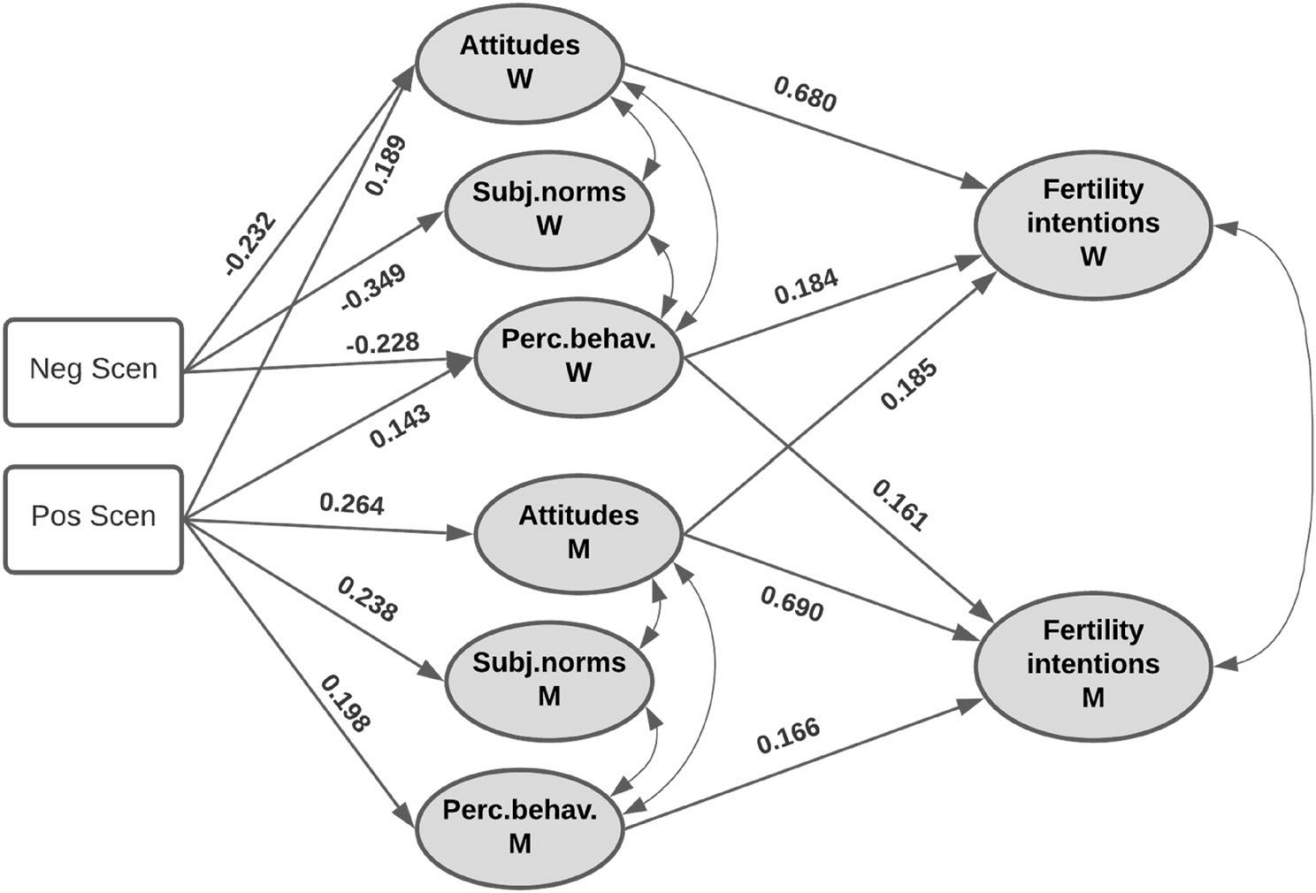
Structural part of the final estimated SEM (variances and covariances omitted for the sake of parsimony)

The final estimated SEM provided an overall good fit to the data (CFI = 0.913, TLI = 0.902, and RMSEA = 0.066). Parameter estimates are displayed in Table 3 for the measurement part and in Table 4 for the structural part of the model (see Fig. 2 for the final model).

**Table 4:** Structural part of the estimated SEM (M = men; W = women)

	Unstandardized coefficients	SE	*z-*value	*P(* >*|z|)*	Standardized coefficients
Women					
Attitude					
Negative scenario	− 0.609	0.156	− 3.913	< 0.001	− 0.232
Positive scenario	0.498	0.141	3.543	< 0.001	0.189
SN					
Negative scenario	− 0.720	0.13	-5.524	< 0.001	− 0.349
PBC					
Negative scenario	− 0.472	0.131	− 3.608	< 0.001	− 0.228
Positive scenario	0.297	0.117	2.542	0.011	0.143
Men					
Attitude					
Positive scenario	0.684	0.143	4.780	< 0.001	0.264
SN					
Positive scenario	0.470	0.129	3.647	< 0.001	0.238
PBC					
Positive scenario	0.382	0.114	3.355	0.001	0.198
Women					
Intention					
Attitude W	1.682	0.188	8.929	< 0.001	0.680
PBC W	0.576	0.236	2.443	0.015	0.184
Attitude M	0.463	0.091	5.101	< 0.001	0.185
Men					
Intention					
PBC W	0.487	0.123	3.962	< 0.001	0.161
Attitude M	1.676	0.181	9.275	< 0.001	0.690
PBC M	0.544	0.232	2.345	0.019	0.166
Covariances					
Intention W–Intention M	0.475	0.218	2.176	0.030	0.203
Attitude W–SN W	0.629	0.086	7.336	< 0.001	0.596
Attitude W–PBC W	0.852	0.09	9.439	< 0.001	0.799
SN W–PBC W	0.534	0.075	7.092	< 0.001	0.630
Attitude M–SN M	0.711	0.093	7.682	< 0.001	0.666
Attitude M–PBC M	0.790	0.091	8.635	< 0.001	0.754
SN M–PBC M	0.500	0.075	6.689	< 0.001	0.622
Attitude items 5–7 W	0.383	0.059	6.518	< 0.001	0.402
Attitude items 5–7 M	0.362	0.056	6.454	< 0.001	0.400
Indirect effects					
Negative scenario to Intention W	− 1.295	0.323	− 4.006	< 0.001	− 0.200
Negative scenario to Intention M	− 0.230	0.085	− 2.713	0.007	− 0.037
Positive scenario to Intention W	1.326	0.301	4.412	< 0.001	0.204
Positive scenario to Intention M	1.355	0.294	4.614	< 0.001	0.215

Only significant coefficients are reported for the sake of clarity

For latent variables representing our constructs, the factor loading of each item was significant (all ps < 0.001). All of our scales showed satisfactory convergent validity and reliability. Indeed, all alpha and CR values were above the threshold of 0.70. The AVE was greater than 0.50 for all scales, except for the subjective norms of men. Nevertheless, convergent validity could also be considered satisfactory in this case due to the AVE value being 0.40 and the CR higher than 0.70 Lam (2012). Given that the validity and reliability of our measures was confirmed, the structural model was tested without any further modification.

According to our findings, perceived economic uncertainty affected both women and men’s attitudes, subjective norms, and perceived behavioral control, thus confirming Hypothesis 1. Nevertheless, some relevant differences could be observed between women and men. For women, compared to the control group, the negative scenario had significant effects on attitudes, subjective norms, and perceived behavioral control, so that high economic uncertainty was associated with less positive attitudes, less favorable subjective norms, and lower perceptions of control over fertility. In contrast, when compared to the control group, the negative scenario affected none of these variables for men, suggesting that their attitudes, subjective norms, and perceived behavioral control did not change upon being induced to imagine a highly uncertain economic future. Differently, the positive scenario, compared to the control group, affected both sexes (only its effect on women’s subjective norms was not significant), which suggests that imagining a future with low economic uncertainty could improve both couple members’ beliefs about having a child in the next three years.

This model also allowed us to test our mediational Hypothesis 3 (H3), according to which both partners’ attitudes, subjective norms, and perceived behavioral control might be responsible for the relationship between economic uncertainty and fertility intentions of the couple members. As expected, women and men’s fertility intentions were significantly correlated. As we observed above (see Table 3), the attitudes, subjective norms, and perceived behavioral control significantly covaried among both women and men. Interestingly, all women’s variables were significantly correlated with all men’s variables. Table 4 displays covariance values; as they were all significant, this confirms that attitudes, subjective norms, and perceived behavioral control are interrelated factors. Significant indirect effects of our mediators on the dependent variables indicated full mediation, in support of H3. In line with our prediction, the indirect effects of the scenarios on women’s fertility intentions were significant (negative scenario, indirect effect = − 0.20, p < 0.001; positive scenario, indirect effect = 0.20, p < 0.001). As shown in Fig. 2, women’s attitudes and perceived behavioral control mediated the relationship between high perceived economic uncertainty and fertility intention, while the relationship between low perceived economic uncertainty and women’s fertility intention was mediated not only by women’s own attitudes and perceived behavioral control, but also by the attitudes of their partners.

The indirect effects also were significant for men, though the effect of the negative scenario (high perceived economic uncertainty) was weaker than the effect of the positive one (negative scenario, indirect effect = − 0.04, p < 0.01; positive scenario, indirect effect = 0.21, p < 0.001). The relationship between high perceived economic uncertainty and men’s intention to have a child was mediated by their partner’s perceived behavioral control. In contrast, low economic uncertainty affected men’s fertility intentions via the mediation of their own attitude, and through their own and their partner’s perceived behavioral control.

Partially in line with Hypothesis 2, we observed some significant actor and partner effects. Women’s intention to have a child was predicted by their own attitudes and perceived behavioral control (actor effects): increased levels of the two were associated with a higher intention to have a child in the next three years. Interestingly, men’s attitudes toward having a child were a significant predictor of women’s intentions, so that women’s fertility intentions were higher when their partner presented more favorable childbearing attitudes (partner effect).

Men’s fertility intentions were predicted by their own attitudes and perceived behavioral control (actor effects): as observed for women, more favorable attitudes and greater perceived behavioral control were associated with higher intentions to have a child in the next 3 years. Again, we observed a relevant partner effect, as women’s perceived behavioral control was significantly associated with their partners’ fertility intention: the more women perceived their ability to control their fertility behavior, the more men intended to have a child in the next three years. However, we found that men and women’s subjective norms were not associated with either their own or their partners’ fertility intentions. The model explained a comparatively high percentage of variance in both women (76.1%) and men’s (72.4%) fertility intentions.

Despite not representing an aim of our research, on the basis of the results obtained, we decided to further examine how perceived economic uncertainty could be related to discrepancies in within-couple fertility plans. We tested another model in which the economic scenario was posited as the independent variable, discrepancies in the couple members’ attitudes, subjective norms, and perceived behavioral control as the mediating variables, and fertility intention discrepancies as the dependent variable (which we computed as differences between the female and male partner).

**Fig. 3:**
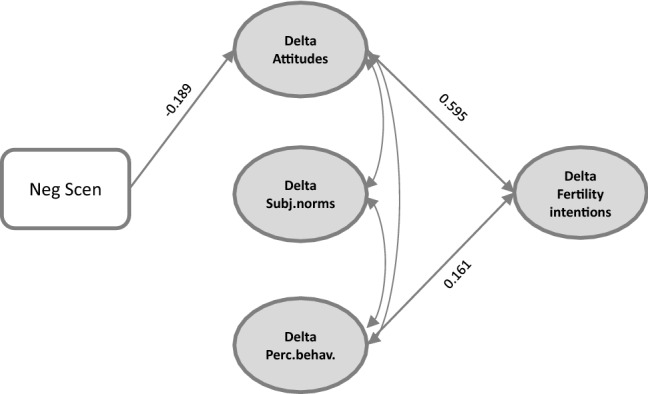
Predictors of fertility intentions' discrepancies within the couple. Deltas represent differences between the female and the male partner (women’s score minus men’s score)

This model showed a good fit to the data (CFI = 0.942, TLI = 0.930, and RMSEA = 0.057). As displayed in Fig. 3, only the negative scenario produced differences in the two partners’ attitudes, which in turn were associated with differences in their intentions to have a child in the next three years.

## Discussion

Contemporary demographic research on the effects of economic uncertainty on fertility intentions is surprisingly silent on the major psychological factors which affect reproductive decisions, and often lacks a couple perspective in which not only women, but also men’s fertility intentions are examined directly (Stykes, 2018; Waller & Bitler, 2008). This study contributes to the literature by highlighting the importance of the psychological processes behind reproductive behaviors, and by examining both partners’ perspectives on fertility. We analyzed how perceiving a negative or positive economic future might affect both couple members’ fertility plans. To this end, we proposed a dyadic extension (Lenne et al., 2019) of the TPB (Ajzen, 1991) to account not only for the psychological processes that might link perceived economic uncertainty to individuals’ fertility intentions, but also for the reciprocal influences possibly present within couples. Such an approach allowed us to conceptualize childbearing as the outcome of a joint couple decision-making process (Stein et al., 2014).

Our study has yielded experimental evidence for the impact of perceived economic uncertainty on fertility intentions among both female and male partners of the same couple. In line with previous studies adopting a gender perspective (Novelli et al., 2021), we detected certain differences between women and men. Indeed, women’s fertility plans varied depending on the perception of both low and high economic uncertainty, meaning that their attitudes towards having a child were more negative when they perceived economic uncertainty to be high, and more positive when it appeared to be low. Men were also more favorable toward fertility when perceived economic uncertainty was low; however, their attitudes, subjective norms and perceived behavioral control were not influenced by high perceived economic uncertainty. These findings suggest that women are more cautious than their partners when they perceive the threat of an uncertain economic context, which might discourage their fertility plans. In contrast, both partners’ attitudes towards fertility were more favourable in the context of a bright economic future. As revealed by further analyses on within-couple discrepancies, the negative scenario could affect differences in the two members’ attitudes and fertility intentions, favoring situations in which the female partner displays negative fertility intentions, while the male partner projects positive ones.

A dyadic extension of the TPB (Ajzen, 1991; Lenne et al., 2019) proved to be useful in explaining fertility intentions. Assortative mating and partnerships homogamy within couples represent topics of growing demographic interest in light of their implications for family dynamics (Esteve et al., 2016; Nitsche et al., 2018; Van Bavel, 2017; Van Bavel et al., 2018). Accordingly, both partners’ attitudes, subjective norms, and perceived behavioral control were affected by the perception of economic uncertainty, and nearly all of these factors played a specific role in predicting women and men’s fertility intentions. In line with prior research, we found attitudes toward having a child to be the strongest predictors of intentions (Ajzen & Klobas, 2013). Perceived behavioral control was also found to have a significant role. Given that reproductive behavior cannot be completely deliberative, the perception of control over such behavior is crucial in forming intentions, in line with the TPB’s theoretical assumptions (Ajzen, 1991).

However, subjective norms seem to have no significant impact on fertility intentions. The coefficients associated with one’s parents, friends, and partner were similar, which suggests that all of them contributed to the formation of the participants’ subjective norms. In contrast to previous evidence showing that significant others can have a relevant role in determining one’s fertility plans in Italy (e.g., Rosina & Fabroni, 2004; Vignoli & Salvini, 2014), our findings suggest that fertility intentions are primarily determined by the individual’s own attitudes, rather than by the perceptions of other referents’ expectations.

Notably, for both attitudes and perceived behavioral control, we observed significant partner effects. Women’s decision-making was affected by their partner’s childbearing attitudes; when their partner favorably evaluated the idea of having a child in the next three years, women’s fertility intentions tended to be higher. These findings suggest that women are more likely to make fertility plans when both their own and their partner’s attitudes toward having a child are positive, which accords with previous studies showing that couples with similar attitudes have a higher chance of transitioning to parenthood (e.g., Hudde & Engelhardt, 2020). For men, what emerged as especially important was their partner’s perceived behavioral control. The more female partners showed high levels of control over having a child in the next three years, the higher men’s fertility intentions.

Importantly, partners’ attitudes emerged as especially crucial in shaping women’s fertility intentions when perceived economic uncertainty was low, although it did not mediate the relationship between high economic uncertainty and women’s intentions to have a child. Given that women appeared to be especially troubled by a pessimistic economic future, it could be that, in this case, they wish to maintain a higher control over this decision. When the economic future appears encouraging, women might be more willing to consider not only their own attitudes, but also those of their partners when making fertility decisions. In contrast, men were influenced by their partners both in the low and high economic uncertainty conditions. In this case, their partners’ perceived behavioral control mediated the relationship between the two uncertainty conditions and men’s fertility intentions. Indeed, it seems that men are more likely to decide to have a child when they think that their partner is able to control this behavior, as if they attribute the possibility of planning such an event based on women’s ability to control its outcome. When the economic prospects appear positive, they also consider their own attitudes and control over reproductive behavior. Given that the birth of a child has a stronger impact on a woman’s life than on a man’s, men might be more likely to consider their female partner’s point of view in forming their fertility intentions (Testa et al., 2011).

In general, the model explained a comparatively high percentage of variance in both women (76.1%) and men’s (72.4%) fertility intentions, suggesting that fertility plans can be successfully predicted by perceived economic uncertainty and the constructs of the TPB, in line with previous correlational findings (e.g., Ajzen & Klobas, 2013; Baroni et al., 2019). Notably, attitudes and perceived behavioral control fully mediated the relationship between perceived economic uncertainty and fertility intentions, suggesting that these psychological processes are pivotal in the childbearing decision-making process, and should thus be carefully considered in fertility research.

This study has some limitations. First, we only measured intentions, and did not administer a long-term follow-up questionnaire which could allow us to assess effective behavior. Second, our experiment was conducted in one central Italian city, meaning that our findings cannot be generalized to the entire population. Third, the participants were given an economic incentive for their participation, which may have induced a selection bias. Fourth, we did not assess the extent to which the participants effectively believed that the scenario they were presented with was true. Fifth, we collected self-reported data, which can be affected by certain biases. Nonetheless, the between-subject experimental design adopted here allowed us to reduce the social desirability bias, as it was impossible for participants to make comparisons between the different scenarios and answer accordingly. Sixth, we assessed fertility intentions without considering whether the respondent wanted to have children or preferred to remain childless. Future studies could consider both the wanting and timing component, in line with some previous research (e.g., Olafsdottir et al., 2011). Finally, despite the robustness of our findings, we should acknowledge that we cannot establish a causal link between individuals’ attitudes and their partner’s fertility intentions. Further research is needed to establish whether fertility intentions are determined by the couple, or if the couple originally matched on fertility intentions, among other factors.

In spite of these limitations, our study’s experimental design and analytical approach provide strong evidence that individuals’ perceptions of the economic future play a relevant role in their childbearing decision-making processes. Moreover, the results confirm that fertility intentions should be examined by adopting a dyadic perspective, as individuals’ fertility intentions are affected not only by their own beliefs, but also by those of their partners.

In terms of practical implications, results of our study indicate that the presentation of a country’s current and future economic situation in the media can affect childbearing decisions of individuals and couples. Indeed, our findings suggest that presenting scenarios characterized by high economic uncertainty might discourage women from planning a pregnancy, by affecting their attitudes, subjective norms, and perceived behavioral control. Alternatively, more optimistic forecasts could encourage fertility plans among both women and men. This is especially important nowadays, as the COVID-19 pandemic has produced high levels of uncertainty (Gieseck & Rujin, 2020), operating as a multiplier of uncertainty (Egidi & Manfredi, 2021; Luppi et al., 2021). The COVID-19 pandemic, and its disruptive effects on multiple domains of life, have radically changed the European scenario for the following years. Embedded in this contemporary setting, fertility decisions are thus taken in a condition of rising uncertainty: as the future is less predictable, decisions are less based on individuals’ forecasting capacity (Guetto et al., 2022). Under such conditions, the way in which the future is depicted by the media, or by decision-makers through the media, and consequently perceived by individuals in couples, might significantly affect their decisions about having a child in the near future.

## Data Availability

The data that support the findings of this study are available from the corresponding author upon reasonable request.

## Appendix 1

Negative (a) and positive (b) scenarios.
